# Altered Medial Prefrontal Connectivity in Parkinson's Disease Patients with Somatic Symptoms

**DOI:** 10.1002/mds.29187

**Published:** 2022-08-22

**Authors:** Stefano Delli Pizzi, Raffaella Franciotti, Piero Chiacchiaretta, Antonio Ferretti, Richard A. Edden, Carlo Sestieri, Mirella Russo, Stefano L. Sensi, Marco Onofrj

**Affiliations:** ^1^ Department of Neuroscience, Imaging, and Clinical Sciences University G. d'Annunzio of Chieti‐Pescara Chieti Italy; ^2^ Institute for Advanced Biomedical Technologies (ITAB), University G. d’Annunzio of Chieti‐ Pescara Chieti Italy; ^3^ Service of Molecular Neurology, Center for Advanced Studies and Technology (CAST) University G. d’Annunzio of Chieti‐ Pescara Chieti Italy; ^4^ Advanced Computing Core, Center for Advanced Studies and Technology (CAST) University G. d’Annunzio of Chieti ‐ Pescara Chieti Italy; ^5^ Department of Advanced Technologies in Medicine & Dentistry University G. d’Annunzio of Chieti ‐ Pescara Chieti 66100 Italy; ^6^ Russell H. Morgan Department of Radiology The Johns Hopkins University School of Medicine Baltimore Maryland USA; ^7^ F.M. Kirby Center for Functional MRI Kennedy Krieger Institute Baltimore Maryland USA

**Keywords:** fMRI, GABA, medial prefrontal cortex, Parkinson's disease, somatic symptom disorder

## Abstract

**Background:**

The high co‐occurrence of somatic symptom disorder (SSD) in Parkinson's disease (PD) patients suggests overlapping pathophysiology. However, little is known about the neural correlates of SSD and their possible interactions with PD. Existing studies have shown that SSD is associated with reduced task‐evoked activity in the medial prefrontal cortex (mPFC), a central node of the default‐mode network (DMN). SSD is also associated with abnormal γ‐aminobutyric acid (GABA) content, a marker of local inhibitory tone and regional hypoactivity, in the same area when SSD co‐occurs with PD.

**Objectives:**

To disentangle the individual and shared effects of SSD and PD on mPFC neurotransmission and connectivity patterns and help disclose the neural mechanisms of comorbidity in the PD population.

**Methods:**

The study cohort included 18 PD patients with SSD (PD + SSD), 18 PD patients, 13 SSD patients who did not exhibit neurologic disorders, and 17 healthy subjects (HC). Proton magnetic resonance (MR) spectroscopy evaluated GABA levels within a volume of interest centered on the mPFC. Resting‐state functional MR imaging investigated the region's functional connectivity patterns.

**Results:**

Compared to HC or PD groups, the mPFC of SSD subjects exhibited higher GABA levels and connectivity. Higher mPFC connectivity involved DMN regions in SSD patients without PD and regions of the executive and attentional networks (EAN) in patients with PD comorbidity.

**Conclusions:**

Aberrant reconfigurations of connectivity patterns between the mPFC and the EAN are distinct features of the PD + SSD comorbidity. © 2022 The Authors. *Movement Disorders* published by Wiley Periodicals LLC on behalf of International Parkinson and Movement Disorder Society

The somatic symptom disorder (SSD) is a neuropsychiatric condition characterized by excessive attention to physical symptoms that cause exaggerated concerns and emotional distress and, sometimes, highly disabling consequences.^1^ The SSD is a highly prevalent comorbidity in Parkinson's disease (PD).^2^, ^3^, ^4^, ^5^, ^6^, ^7^ Depending on the country and medical setting, the frequency of SSD ranges from 3.5% to 18.4%,^8^ in the general population, and from 7.0% to 66.7%^9^, ^10^, ^11^ in PD patients.

The high co‐occurrence of SSD in PD patients raises the possibility of overlapping pathophysiology. However, little is known about the neural correlates of SSD and their possible interaction with PD. Several studies have shown that the reduced activity of the medial prefrontal cortex (mPFC)—a region involved in reality monitoring,^12^ emotional valence, and introspective processing of sensory information^13^—plays a pathophysiologic role in SSD.^8^, ^14^, ^15^, ^16^, ^17^ The mPFC is the anterior hub of the default mode network (DMN), a set of brain regions activated by internally oriented cognition, such as self‐related processing and introspection,^18^ and deactivated by externally oriented tasks.^19^ Moreover, the activity of the mPFC promotes the crosstalk and synergic integration of the DMN with the (frontoparietal) executive and (dorsal) attentional networks (EAN).^20^, ^21^, ^22^ These are brain regions that are activated by externally oriented tasks and show a typical pattern of anticorrelation with the DMN.^23^ The DMN‐EAN balance modulates a wide range of cognitive domains, including the discrimination and filtering of significant peripheral stimuli, the integration of emotion‐ and memory‐ introspective information, and the cognitive representations of salient stimuli.^24^, ^25^, ^26^, ^27^, ^28^ Recently, we have reported that increased γ‐aminobutyric acid (GABA) content in mPFC is a neural feature of SSD, regardless of PD comorbidity. It has also been proposed that GABA levels within the mPFC are critical to shaping the strength of its functional connectivity in healthy subjects.^29^, ^30^, ^31^, ^32^ However, it is currently unknown whether the altered inhibitory neurotransmission observed in SSD is associated with altered patterns of mPFC functional connectivity and whether these effects are similarly found in SSD patients with or without PD.

The central hypothesis of our study is that SSD patients show dysfunctional neurotransmission within the mPFC and a functional network reconfiguration between the DMN and the EAN. We also test the presence of shared and individual effects of SSD and PD on mPFC neurotransmission and connectivity patterns that help explain the neural mechanisms of comorbidity in PD patients. To test this hypothesis, we used multimodal magnetic resonance imaging (MRI) to assess neurochemistry changes and variations of mPFC functioning. Proton MR spectroscopy (^1^H‐MRS) was used to measure the contents of GABA and glutamate + glutamine (Glx) within a volume of interest (VOI) centered on the mPFC. Resting‐state functional MRI (rs‐fMRI) was used to investigate functional connectivity between the ^1^H‐MRS VOI and the rest of the brain.

## Material and Methods

### Study Sample

This study was approved by the local institutional ethics committee (protocol n. 1665) and was performed according to the Declaration of Helsinki and subsequent revisions. The study cohort included 18 PD patients with SSD (PD + SSD), 18 PD patients (PD), 13 SSD patients who did not exhibit any other neurological comorbidity in a 5–7 years follow‐up (SSD), and 17 healthy control (HC) subjects. All participants gave written informed consent and were enrolled at the Neurology Clinic of the University “G. d'Annunzio” of Chieti‐Pescara, Italy.

The United Kingdom (UK) Brain Bank Criteria were used for the diagnosis of PD.^33^ The Diagnostic and Statistical Manual of Mental Disorders, Fifth Edition (DSM‐5), and patient interviews were used to evaluate the mental status and the presence of SSD, respectively. The presence and severity of extrapyramidal signs were assessed using the Unified Parkinson's Disease Rating Scale Part III (UPDRS‐III)^34^ and the Hoehn and Yahr (H&Y)^35^ scale. The Mini‐Mental State Examination (MMSE)^36^ and the Frontal Assessment Battery (FAB)^37^ evaluated global cognition and frontal functioning, respectively. Dopamine transporter single‐photon emission computed tomography (DaT‐SPECT) exams were performed only in PD patients to confirm the diagnosis by assessing impaired nigrostriatal dopaminergic transmission. All subjects underwent a preliminary CT/structural MRI brain scan to exclude any factor (eg, lacunar strokes of the basal ganglia or other brain lesions) that could have confounded the clinical outlook. PD patients were treated with doses of dopamine‐mimetic drugs. PD drugs were withdrawn the day of MRI scans and reintroduced after the MRI acquisition. SSD subjects were medication naïve. Exclusion criteria included prior history of major medical conditions, head injury, psychiatric or neurological diseases, such as prodromal or clinical stages of dementia, history of substance abuse, contraindications to the use of MRI, and treatment with anxiolytic or anti‐depressive drugs. Cognitively HC subjects were additionally assessed for: (1) attention skills, sustained attention, divided attention, task coordination, and set‐shifting using the Trail Making Test; (2) selective attention using attentional matrices; (3) verbal short‐term and long‐term memory using the Babcock Story Recall Test; and (4) auditory working memory using the forward and backward digit span tests.

### Evaluation of SSD

All participants underwent semi‐structured interviews performed by a rater blinded to SSD diagnosis. The interviews were structured on the DSM‐5 and assessed somatic complaints using examples and a checklist exhibited to patients and caregivers.^1^ The interviews investigated the presence of SSD traits (ie, dependency, mannerism, viscosity, adoption of a sick role, and histrionic and dramatic representation of illness). Past SSD was also assessed by considering information from prior hospital records and reports from the patient's general practitioner acquired in the previous 4 to 20 years. Methods regarding the SSD categorization are reported in the Supporting Data. The Neuropsychiatry Inventory assessed the presence of somatic‐type delusional disorders. Patients were also tested with the symptom questionnaire. The Diagnostic Criteria for Psychosomatic Research were used to ensure a parametric assessment of the symptoms in a neurodegenerative condition.

### MRI Protocol

MRI data were collected with a Philips Achieva 3 Tesla scanner (Philips Medical Systems, Best, The Netherlands). Structural images were acquired using a 3‐dimensional T_1_‐weighted turbo field‐echo sequence (repetition time/echo time [TR/TE] = 11/5 ms, slice thickness of 0.8 mm). T_2_‐weighted fluid attenuation inversion recovery images were also acquired to perform a neuroradiological evaluation.

**FIG 1:**
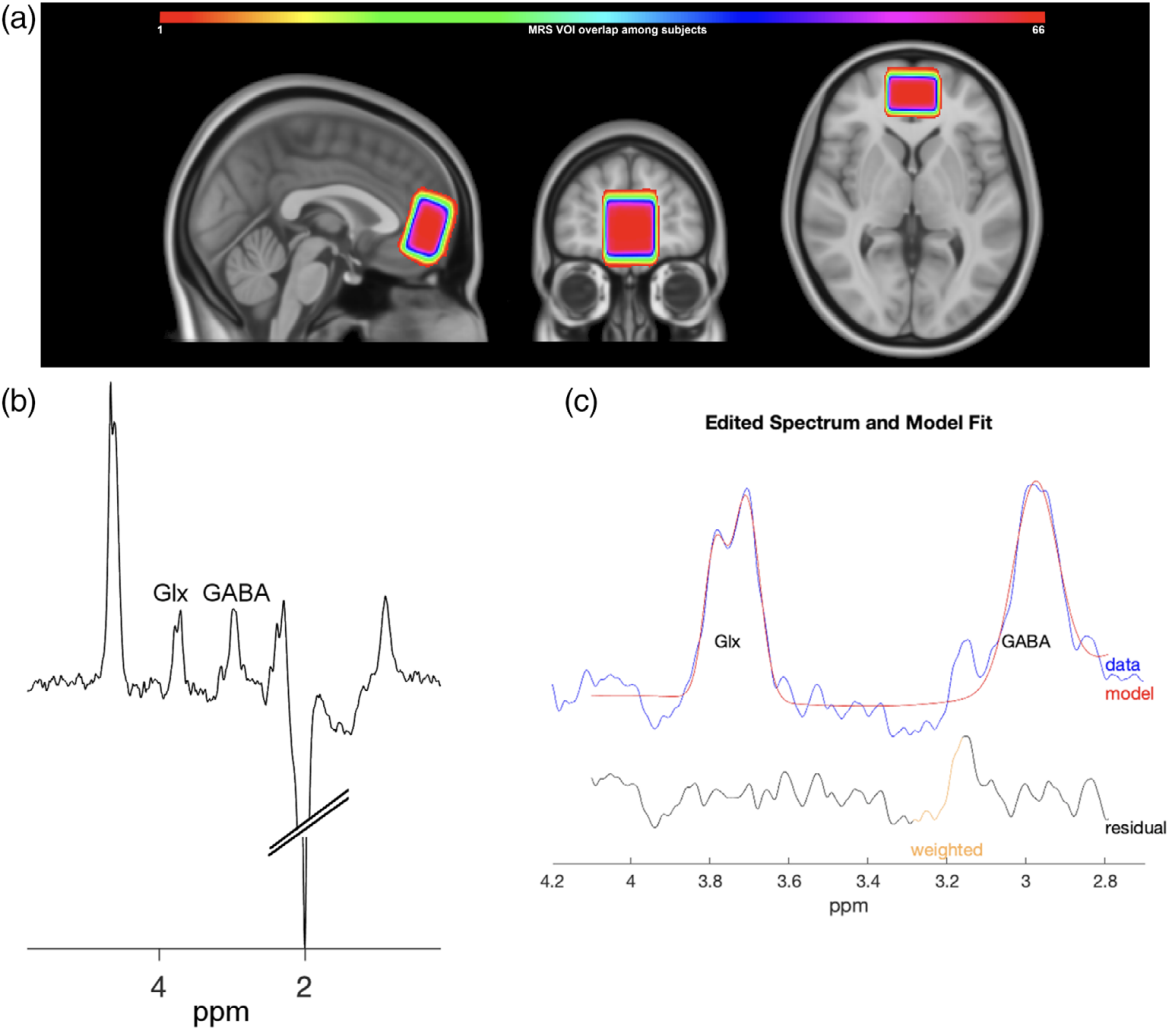
Proton magnetic resonance spectroscopy (^1^H‐MRS). (**A**) Depicts the location of all ^1^H‐MRS VOI of 2.0 (anterior–posterior) × 3.0 (left–right) × 3.0 (craniocaudal) cm^3^ centered on the medial prefrontal cortex of each subject. The density map shows the regional overlaps among participants according to color bars. (**B**) Depicts MR spectra from a representative study subject. (**C**) Shows GABA and Glx peaks from a representative study subject. The representative edited spectrum is depicted in blue, the estimated metabolite model in red, and the residual in black. [Color figure can be viewed at wileyonlinelibrary.com]

A ^1^H‐MRS VOI was centered on the mPFC (Fig. 1A) and acquired by using Mescher‐Garwood point resolved spectroscopy sequence (MEGA‐PRESS).^32^ To maintain a suitable signal‐to‐noise ratio, the ^1^H‐MRS VOI size was 2.0 (anterior–posterior) × 3.0 (left–right) × 3.0 (skull‐caudal) cm^3^. An experienced MRI technician accurately placed the MRS VOI on the mPFC, in line with the seminal study on the DMN by Raichle and colleagues.^19^ The center of the MRS VOI crossed: (1) a line passing along splenium and genus of the corpus callosum on the axial plane; (2) a medial line traversing the septum pellucidum on the coronal plane. Finally, on the sagittal view, the posterior side of the voxel was placed adjacently to the pregenual anterior cingulate cortex; a region not comprised by the voxel given that is part of the limbic system. The signal of GABA results in a substantial co‐edited contribution from macromolecules, and the resulting measure is generally referred to as GABA+. The MEGA‐PRESS (TR/TE = 2000/68 ms, 320 averages) was used to acquire 1024 points within a spectral width of 2000 Hz.

Resting‐state blood oxygen level dependent fMRI data were acquired using gradient‐echo T_2_*‐weighted echo‐planar sequences (matrix size, 64 × 64; in‐plane voxel size, 3.6 mm × 3.6 mm; slice thickness, 5 mm; and TR = 1100 ms). For each subject, two runs of 300 functional volumes consisting of 21 trans‐axial slices were acquired with a TR of 1100 ms. Participants were instructed to lie still and keep their eyes closed during acquisition.

### Structural MRI Analysis

T_1_‐weighted images were analyzed using the “recon‐all ‐all” command line to obtain the automated reconstruction and labeling of cortical and subcortical regions (FreeSurfer 6.0).^38^ Detail on data preprocessing and processing are provided in Supporting Data.

### MRS Analysis

GANNET, a MATLAB‐based tool,^39^ was used to assess GABA+/total creatine (tCr) and Glx/tCr in each spectrum. Default parameters, including frequency and phase correction of time‐resolved data using spectral registration, were used. In the GANNET‐edited spectrum, Glx signal was quantified from the pseudo‐doublet peaks at 3.75 ppm. GANNET‐estimated signal for GABA and Glx is shown in Fig. 1B,C. Gannet was used to mask the ^1^H‐MRS VOI and co‐register it on the anatomical image. Volumes and masks of grey matter (GM) and white matter (WM) within the ^1^H‐MRS VOI were obtained by combining the outputs of “recon‐all” (FreeSurfer) and “fslmaths/fslstats” (FMRIB Software Library) command lines. All the generated images were visually assessed to validate the positioning of the MRS voxel and evaluate the output of tissue segmentation. ^1^H‐MRS outcomes were shown as ratios of metabolites/tCr^40^ because: (1) this quantification exhibits a performance equal to, or better than, water referencing^41^; (2) the tCr concentrations are independent of SSD or PD^32^; (3) tCr‐referenced metabolite values are expected to be less sensitive to changes related to tissue atrophy.^41^ GABA signal‐to‐noise ratio is in the Gannet output and has been compared to the “Big GABA” output as a benchmark.^42^

### Functional MRI Analysis

The GM mask within the ^1^H‐MRS VOI, which encompasses the left and right GM within the mPFC, was used as a “seed region” for FC analysis using FreeSurfer‐Functional Analysis Stream^38^ (http://surfer.nmr.mgh.harvard.edu/fswiki/FsFastFunctionalConnectivityWalkthrou). Preprocessing included motion and slice timing corrections, masking, registration to the structural image, sampling to the surface, and surface smoothing by 5 mm. Surface sampling of time series data was carried out onto the surface of the left and right hemispheres of the “fsaverage” template of FreeSurfer. Therefore, although the time series data were sampled onto fsaverage, the FC seeds were derived from the individual anatomy. Nuisance regressors were obtained for each participant by extracting the echo‐planar imaging (EPI) average time courses within the ventricle mask and the WM mask (considering the top 5 principal components). These regressors and the motion correction parameters were eliminated from the EPI time series. Temporal band‐pass filtering (0.01 < Hz < 0.1) was applied to restrict the analysis on this frequency range. Because of T1 saturation effects, the first four rs‐fMRI time points were discarded from the analysis. Volumes for which the framewise displacement value (FD) is superior to 0.5 mm were removed. The mean signal time course within the seed region was used as a regressor to assess the pattern of FC. Using the “selxavg3‐sess” command, we performed the first‐level analysis (single‐subject analysis), including the computation of the Pearson correlation coefficient (*r*‐value) between the time series of the seed and the other voxels. The obtained correlation maps were then converted to *Z*‐score maps before entering the second‐level analysis (group analysis). The “isxconcat‐sess” command was used to create a “stack” of maps from all study participants. For a qualitative assessment of whole‐brain patterns of mPFC connectivity in each group, the mri_glmfit command was used to calculate voxelwise maps representing correlation values averaged across subjects of each group. Yeo's functional atlas was used to label clusters of between‐group differences or within‐group correlations. For each participant, the global connectivity strength of the ^1^H‐MRS VOI was calculated in the left and right hemispheres by averaging the *Z*‐score from each voxel contained in the brain.

### Statistical Analysis

On the basis of data distribution, parametric analysis of variance (ANOVA) or nonparametric Kruskal‐Wallis tests evaluated group differences in demographic, imaging outcomes, and clinical data. Between the two PD groups, Mann–Whitney test verified the presence of significant differences in the UPDRS‐III, H&Y scores, and disease duration. A χ^2^ test evaluated group differences in sex. A three‐way 2 × 2 × 2 ANOVA (sex, 2 levels [male, female]; PD status, 2 levels [with PD, without PD]; SSD status, 2 levels [with SSD, without SSD]) was applied to test the between‐group differences and interaction effects among the three factors (ie, sex, SSD, and PD). Significant results are shown on statistical maps and adjusted by setting a cluster‐wise threshold at *P*‐corrected <0.05.^43^ To avoid the pitfalls in cluster‐wise based thresholding, we used a voxelwise (cluster‐forming) threshold at *P* < 0.001.^44^ Step‐wise linear regression assessed the within‐group relationships among the functional connectivity strength and GABA levels. The dependent variable was the functional connectivity strength between the ^1^H‐MRS VOI and cortical targets, which were obtained by comparing non‐SDD (HC plus PD groups) and SSD (SSD plus PD + SSD groups) participants. Sex, GABA, and age were included as independent variables. Spearman's correlation was used to investigate the relationships of MRI metrics with Symptom Questionnaire (SQ) scores.

## Results

### Demographics and Clinical Features

The demographic, clinical, and imaging features of the study participants are shown in Table 1. Symptom Questionnaire‐somatic symptoms subscale items of each group are reported in Supplementary Table S1. No significant differences were observed among groups when evaluating age (*F*_3,65_ = 0.334, *P* = 0.801), educational levels (*H* = 5.425, *P* = 0.143), MMSE (*H* = 4.033, *P* = 0.258), and FAB (*H* = 3.181, *P* = 0.365) scores. PD disease duration (*Z* = −1.631, *P* = 0.111), as well as the scores of UPDRS‐III (*Z* = −0.16, *P* = 0.987) and H&Y (*Z* = 0.157, *P* = 0.203) scales, did not differ between PD groups. A significant difference among groups was found for sex (χ^2^ = 3.879, *P* = 0.049), and the PD group had a preponderance of males (14 males vs. 4 females). Fluid‐attenuated inversion recovery (FLAIR)‐image assessments revealed no altered radiological findings among participants.

**TABLE 1:** Demographic and clinical features of the study participants

Variables	HC (*n* = 17)	SSD (*n* = 13)	PD (*n* = 18)	PD + SSD (*n* = 18)
Age (y)	64.6 ± 10.9	63.8 ± 9.1	66.7 ± 7.2	65.9 ± 8.4
Sex (male %)	53%	54%	78%	56%
Educational level (y)	10.1 ± 4.4	11.3 ± 3.3	9.8 ± 5.2	7.9 ± 4.2
MMSE	27.9 ± 1.2	27.4 ± 1.5	26.7 ± 3.7	27.9 ± 3.6
FAB	16.5 ± 1.5	16.2 ± 1.5	14.6 ± 3.6	15.2 ± 2.6
UPDRS‐III	–	–	13.8 ± 6.2	13.6 ± 6.3
H&Y	–	–	1.6 ± 0.5	1.4 ± 0.5
PD duration (y)	–	–	3.7 ± 2.5	4.7 ± 2.1
NPI total	–	–	1.7 ± 1.0	4.9 ± 1.3*
GABA+/tCr	0.083 ± 0.020	0.116 ± 0.026*	0.084 ± 0.020	0.113 ± 0.028*
Glx/tCr	0.078 ± 0.033	0.096 ± 0.029	0.098 ± 0.045	0.092 ± 0.020
GM‐MRS (mm^3^)	10,381 ± 812	10,323 ± 1096	10,224 ± 688	10,144 ± 531

Values are expressed as mean ± standard deviation (SD). Metrics or scores that are significantly different among groups were marked with an asterisk.

Abbreviations: FAB, Frontal Assessment Battery; GABA, γ‐aminobutyric acid; Glx = glumate + glutamine; GM, grey matter; HC, healthy control; H&Y, Hoehn and Yahr; MMSE, Mini‐Mental State Examination; NPI, Neuropsychiatric Inventory; PD, Parkinson's disease patients without somatic symptom disorder; SSD, patients with somatic symptom disorder unaffected by any neurological or other psychiatric condition; PD + SSD, Parkinson's Disease patients with somatic symptom disorder; UPDRS‐III, Unified Parkinson's Disease Rating Scale III.

### Neurochemistry and Regional Homogeneity

The imaging‐based features of the ^1^H‐MRS VOI are shown in Table 1.

The presence of SSD (*P* < 0.001), but not of PD (*P* = 0.909) or sex (*P* = 0.064), increased the levels of GABA+/tCr (*F*_8,65_ = 5.114, *P* < 0.001).

No significant interaction effects among the SSD, PD, and gender were observed on GABA+/tCr.

Age did not significantly affect variations of GABA+/tCr (*P* = 0.936) among groups.

The GM volume within the MRS VOI (*H* = 3.367, *P* = 0.338) and the Glx/Cr levels (*H* = 3.696, *P* = 0.296) did not differ among groups.

### Functional Connectivity

Qualitative analysis of functional connectivity strength (Supplementary Fig. S1A–D) indicated that all the study groups were characterized by a positive coupling of the ^1^H‐MRS VOI with other DMN nodes. In addition, HC subjects exhibited negative coupling between the ^1^H‐MRS VOI and the EAN, as expected. Instead, decreased anticorrelation, both in spatial extent and voxel value, was found in the PD and SSD groups compared to HC. Crucially, the inverse coupling between the DMN and the EAN even disappeared in the PD + SSD group.

**FIG 2:**
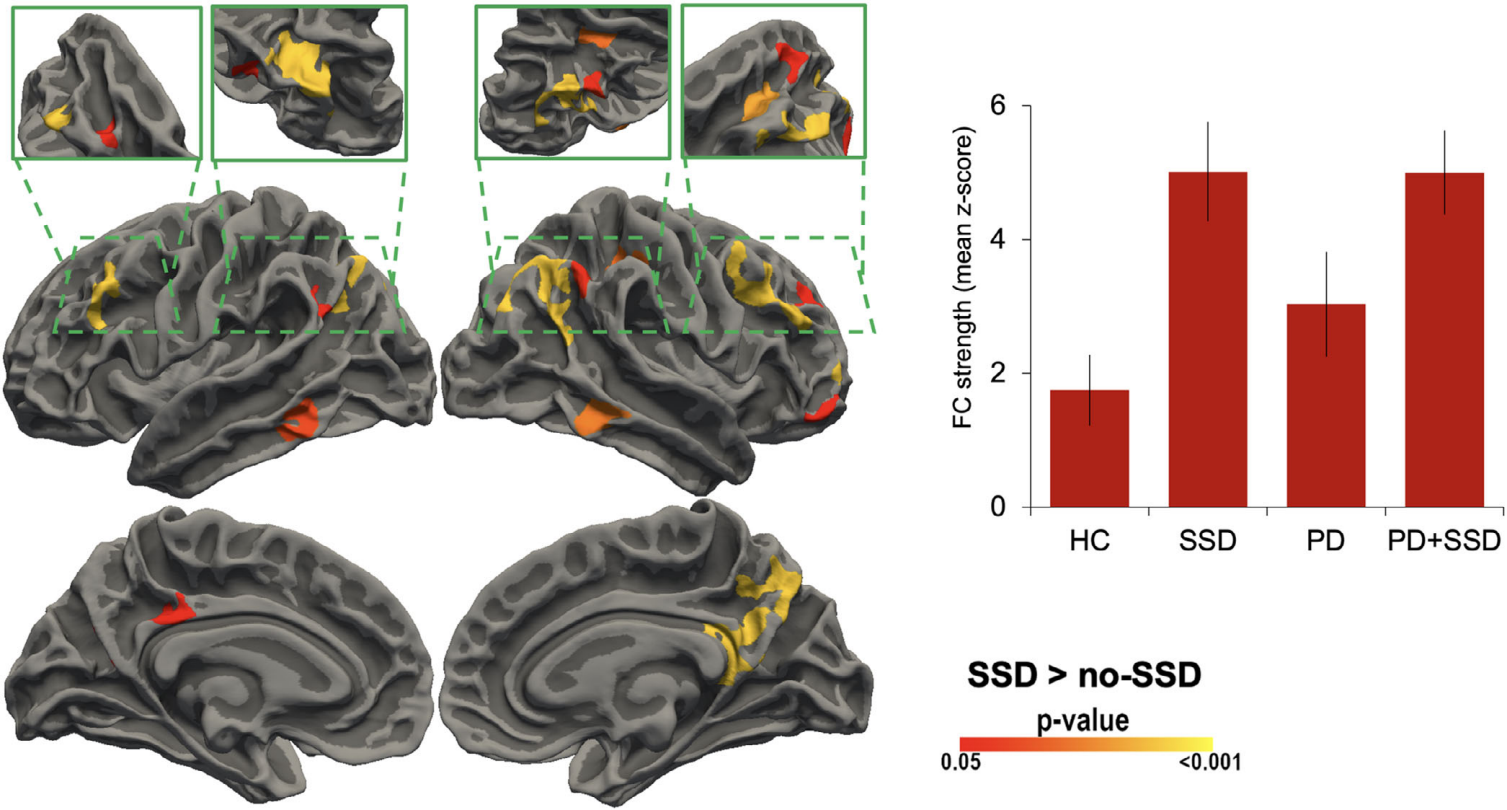
Statistical maps show comparisons of the SSD cohort (SSD patients and PD + SSD patients) with the no‐SSD cohort (HC subjects and PD patients). Clusters changing from red to yellow indicate increased connectivity. The figure depicts regions with a cluster‐wide probability below the corrected *P*‐value of 0.05 and voxelwise (cluster‐forming) threshold at *P* < 0.001. Bar plots show the direction of group averaged values. Abbreviations: HC, healthy controls; PD + SSD, patients with Parkinson's disease without somatic symptom disorder; PD, patients without Parkinson's disease without somatic symptom disorder; SSD, patients with somatic symptom disorder unaffected by any neurological or other psychiatric condition. [Color figure can be viewed at wileyonlinelibrary.com]

Quantitative analyses of functional connectivity identified significant differences and interactions among groups (Supplementary Table S2). The ANOVA indicated a main effect of SSD (Fig. 2). In particular, when compared to the no‐SSD cohort (including both HC and PD), the SSD cohort, with or without PD, exhibited increased mPFC connectivity with core regions of the EAN (ie, lateral PFC, inferior temporal and parietal cortices) and the DMN (ie, precuneus, superior frontal cortex, angular gyrus, and inferior temporal cortex).

**FIG 3:**
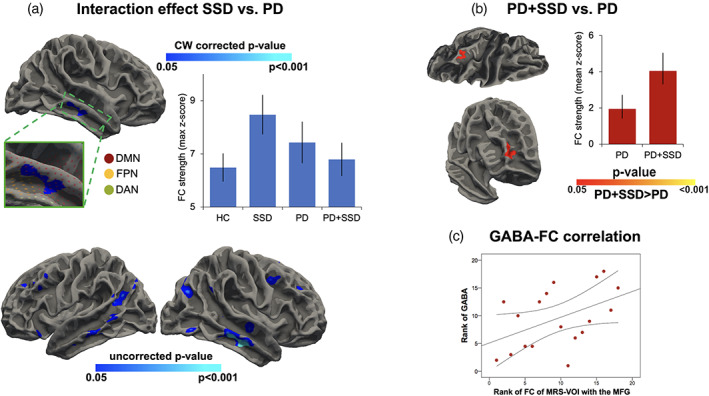
Statistical maps showing the main and interaction effects of PD and SSD on functional connectivity of the ^1^H‐MRS VOI centered on the medial prefrontal cortex. (**A**) Shows the interaction between PD and SSD. The upper panel reports statistically corrected maps. The lower panel reports statistically uncorrected maps. (**B**) Shows the comparison between PD + SSD and PD patients. Clusters changing from red to yellow indicate increased connectivity. Clusters changing from blue to dark blue indicate the brain areas where PD pathology affects the functional reorganization promoted by SSD. The figure depicts regions with a cluster‐wide probability below the corrected *P*‐value of 0.05 and voxel‐wise (cluster‐forming) threshold at *P* < 0.001. (**C**) Shows bar plots that depict the direction of the interaction effect and group averaged values. Abbreviations: DAN, dorsal attention network; DMN, default mode network; FPN, frontoparietal network; HC, healthy controls; MFG, medial frontal gyrus; PD + SSD, patients with Parkinson's disease without somatic symptom disorder; PD, patients without Parkinson's disease without somatic symptom disorder; SSD, patients with somatic symptom disorder unaffected by any neurological or other psychiatric condition. [Color figure can be viewed at wileyonlinelibrary.com]

An interaction effect was observed between PD and SSD on the functional connectivity of the ^1^H‐MRS VOI (Fig. 3), such as the PD plus SSD comorbidity is associated with reduced functional reorganization between the 1H‐MRS VOI and temporal components of the DMN promoted by SSD (Fig. 3A, upper panel). Of note, the uncorrected map indicated the presence of an interaction effect between the SSD and PD also in other components of the DMN, such as the temporoparietal junction and angular gyrus, bilaterally (Fig. 3A, lower panel).

When specifically examining the difference between HC and SSD subjects (without PD), the enhanced connectivity effect was selectively observed in regions of the DMN (ie, angular gyrus, superior frontal, and inferior temporal cortices) (Supplementary Fig. S2A). Instead, compared with PD patients (Fig. 3B) or HC subjects (Supplementary Fig. S2B), PD + SSD patients showed increased functional connectivity with frontal and parietal components of the EAN, but not with the DMN. The comparison of PD + SSD patients with HC subjects also revealed increased connectivity with the inferior frontal cortex within the EAN/limbic system.

In the PD + SSD group, we found that GABA content in the mPFC was significantly associated with connection strength between the mPFC and the right medial frontal region of EAN (model, *F*_3,18_ = 3.302, *P* = 0.05; GABA, β = 0.57, *P* = 0.036; age: *P* = 0.107; sex, *P* = 0.671) (Fig. 3C). No significant correlations were found between GABA levels and long‐range connection strength in the PD and HC groups.

There was no significant correlation between the SQ test scores and the MRI metrics.

Additional control analyses revealed that no influence of SSD, PD, and sex on the global connectivity (*F*_8,65_ = 1566, *P* = 0.156). Moreover, the head movement amplitude (measured with the FD) did not differ between the four groups (*F*_3,65_ = 0.924, *P* = 0.435). Finally, no significant changes or interaction effects in cortical thickness were found among groups.

## Discussion

In the present study, we investigated the neurochemical properties and functional connectivity of the mPFC in separate groups of SSD + PD, PD, SSD, and HC participants. In line with our previous ^1^H‐MRS study, SSD cohort (including SSD and PD + SSD patients) exhibited higher GABA+ levels within the mPFC. Therefore, the present study indicates that the GABAergic imbalance appears to be a general feature of SSD, independently from PD comorbidity. Moreover, the SSD cohort showed distinct patterns of enhanced functional connectivity of the mPFC with different networks, depending on the presence or absence of PD comorbidity. Whereas SSD patients increased connectivity with other regions of the DMN, SSD + PD subjects exhibited increased connectivity mainly with areas of the EAN.

GABA plays a crucial role in shaping cognitive functioning. Recent evidence, combining fMRI and ^1^H‐MRS data, suggests that the imbalance of the GABAergic system alters the cortical excitatory/inhibitory equilibrium and could generate maladaptive functional network interactions. These changes provide a shared pathological framework for several neuropsychiatric conditions like anxiety, depression, and psychosis.^29^, ^30^, ^45^, ^46^

^1^H‐MRS is a non‐invasive tool that quantifies GABA levels in the brain. It is worth noting that the ^1^H‐MRS signal detects total, rather than synaptic, GABA levels,^47^, ^48^ thereby providing quantitative assessments of the overall inhibitory tone within a region of interest.^49^, ^50^, ^51^ Therefore, high GABA levels are likely associated with a regional increase of the inhibitory tone, and then its altered content indicates alterations within the prefrontal area that could be linked to dysfunctional activity.^52^, ^53^

In line with this notion, in the SSD patients, we found increased mPFC functional connectivity with the DMN and frontoparietal components of the EAN (summarized in Fig. 2). This phenomenon might serve as a defective adaptive role of the mPFC. This is in analogy with maladaptive plasticity processes observed in the context of several neurodegenerative conditions or acute brain lesions.^54^, ^55^, ^56^ We speculate that the enhanced connectivity may become disadvantageous because the process negatively impinges on the brain's modular organization. In particular, the mechanism may lead to an excessive integration among DMN components involved in introspective processes and pathological rumination as observed in major depression^57^, ^58^ and conversion disorders.^2^, ^59^, ^60^ Moreover, the increased connectivity of the mPFC with the EAN may impair the physiological anticorrelation typically observed between task‐positive (eg, EAN) and task‐negative (eg, DMN) networks,^61^, ^62^ affecting the capacity to discriminate the internal state from the external environment.^63^, ^64^

**FIG 4:**
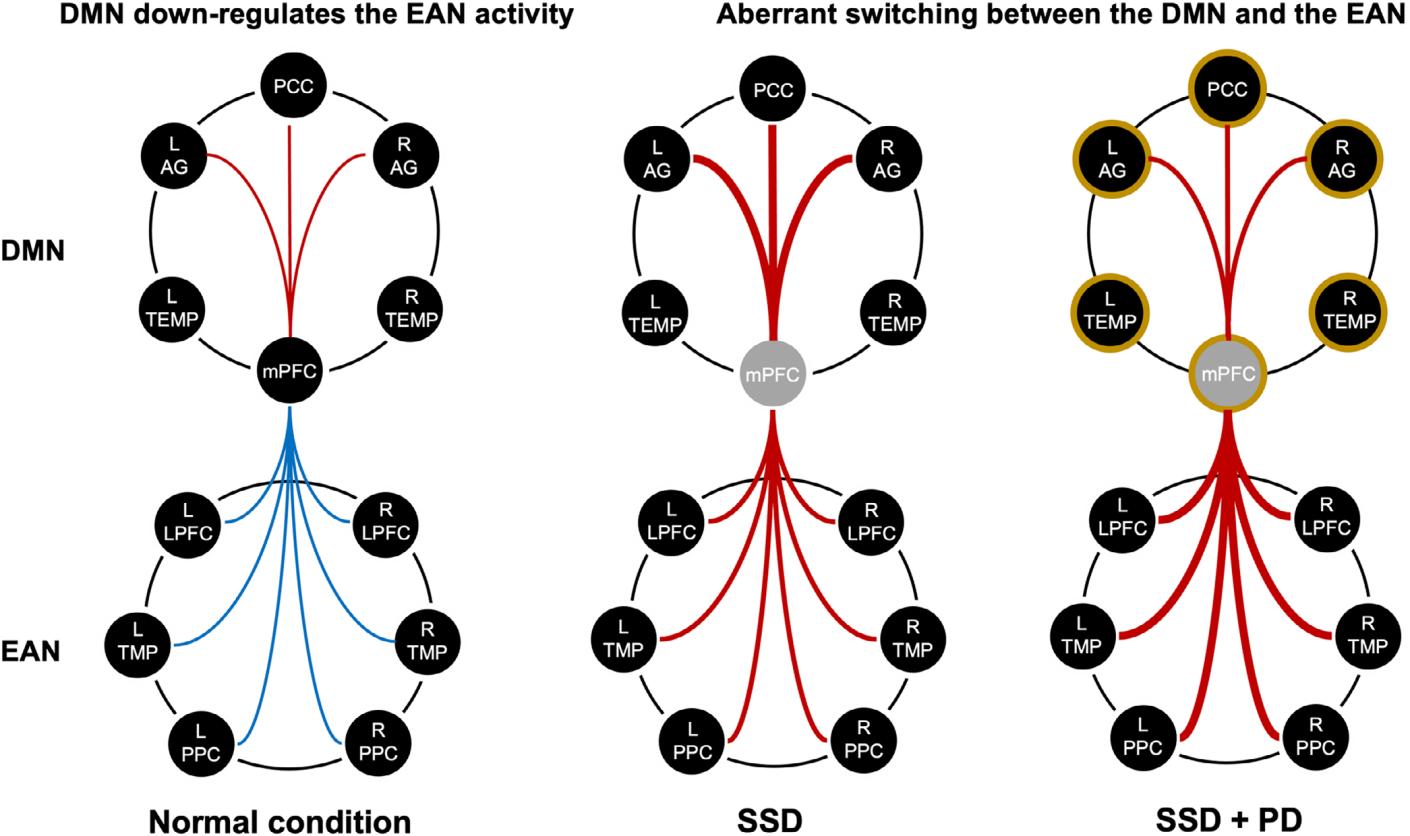
Proposed model for the production of somatic symptom disorder in patients with or without Parkinson's disease. Under normal conditions, the DMN is a central orchestrator for information integration and routing. The DMN downregulates the EAN activity, reducing goal‐irrelevant processing and preventing an overflow of poorly managed information. Therefore, the mPFC is correlated with other DMN regions and anticorrelated with EAN regions. In SSD patients, prefrontal dysfunctions may lead to excessive integration between DMN components involved in introspective processes and pathological rumination observed in major depression. In PD patients with SSD, the DMN is one of the first cortical networks affected by PD pathology as α‐synuclein spreads from subcortical structures to the medial prefrontal areas and subsequently across the remaining parts of the neocortex.^66^ In this context, the spreading of PD pathology may mitigate the adaptive processes within the DMN. The reconfiguration of mPFC connectivity could spill over to the EAN, exacerbating the loss of functional anticorrelation between the DMN and EAN and generating a failure in assessing internal states versus the external environment. Abbreviations: AEN, attentional/executive network; DMN, default‐mode network; HC, healthy controls; L, left; PD + SSD, patients with Parkinson's disease without somatic symptom disorder; PD, patients without Parkinson's disease without somatic symptom disorder; R, right; SSD, patients with somatic symptom disorder unaffected by any neurological or other psychiatric condition. Red and blue lines represent the positive or negative functional coupling, respectively. The line thickness indicates the strength of functional connections. The grey sphere depicts increased inhibitory tone in the mPFC. The brown boundaries highlight areas primarily affected by the spreading of PD pathology. [Color figure can be viewed at wileyonlinelibrary.com]

This study also investigated specific connectivity features that may provide new insights into the pathophysiology of SSD in PD. On a speculative note, our data suggest that the aberrant reconfiguration of connectivity patterns across the EAN is a distinct feature of SSD + PD patients, as indicated by the interaction effect. The DMN is among the first cortical networks affected by PD pathology as α‐synuclein spreads from subcortical structures to the medial prefrontal areas and only subsequently across the remaining neocortical DMN components.^65^, ^66^ The spreading of synuclein loads may decrease adaptive processes within the DMN. In this context, the reconfiguration of mPFC connectivity involving the EAN could likely exacerbate the loss of functional anticorrelation m between the DMN and EAN (Fig. 4). However, further investigation with empirical data is needed to test of this theoretical model.

This study presents some limitations. First, a further longitudinal/prospective study involving a larger cohort of PD patients is needed to understand how/whether functional connectivity changes promote SSD in PD patients. Moreover, the current study design does not permit testing causality and it is not possible to ascertain if GABAergic alterations cause functional connectivity changes or vice‐versa. Second, the neuropsychological evaluations administered to the PD patients were brief. Nevertheless, PD patients underwent cognitive screening tests to exclude PD‐Mild Cognitive Impairment (MCI). In a few cases, patients were also assessed by more in‐depth neuropsychological evaluations that ruled out the presence of MCI and confirmed a normal global cognition. Third, the sample size, especially for the SSD cohort, was relatively small for a cross‐sectional rs‐fMRI study. However, we would like to stress that this is the first multimodal MRI protocol that combines high‐resolution MRI, proton MR spectroscopy, and rs‐fMRI in SSD and PD subjects. The application of such a multimodal MRI protocol to a clinical population is very complex. It requires higher degrees of compliance by participants because of the long duration of scan time. Furthermore, we opted for an experimental design that included clinically selected cohorts, including patients with a de novo diagnosis of SSD. Fourth, the orbitofrontal cortex is an area known to be sensitive to EPI geometrical deformations. To address this potential issue, slice angulation was optimized to minimize the impact of in‐plane susceptibility gradients and reduce EPI distortions during data acquisition. Moreover, parallel imaging (sensitivity encoding [SENSE] factor = 1.8) was used to shorten the readout time. We recognize that B_0_ field maps better correct severe distortions. However, visual inspections did not reveal any significant misalignment issues after EPI‐T_1_ co‐registration in our data.

In conclusion, increased GABA levels were found in SSD patients with and without PD. Aberrant reconfigurations of connectivity patterns across the executive/attentional network and the absence of hyper‐connectivity with the DMN are distinct features of PD patients who also exhibit SSD. Our results and the current experimental evidence support the notion that longitudinal assessments are needed to verify whether specific mPFC neurochemical and functional alterations exhibited by SSD‐PD subjects can be used to predict worsening of clinical conditions or incoming dementia.

## Financial Disclosures

S.D.P. serves as academic editor of Scientific Reports, Behavioral and Brain Functions, and Medicine. S.D.P. was supported by research funding from Search for Excellence program from UdA. M.O. has served on the scientific advisory boards of GlaxoSmithKline, Novartis, Lundbeck, Eisai, Valeant, Medtronic, and Newron; has received speaker honoraria from Zambon, the World Parkinson Congress, the Movement Disorder Society, and the Atypical Dementias congress; publishing royalties from Springer; was an invited guest and lecturer for the Mental Disorders in Parkinson Disease Congress; serves on the editorial board of Medicine (Baltimore) and Frontiers in Neurology; has been employed as a speaker for Boehringer Ingelheim, GlaxoSmithKline, UCB, and Zambon; and has received research support from the Italian Ministry of Health and the Italian Ministry of Education. S.L.S. serves as associate editor of Frontiers in Neuroscience, Frontiers in Psychiatry, PlosOne, and Scientific Reports. He is supported by non‐profit agencies (the Italian Ministry of Health, the AIRAlzh Onlus [ANCC‐COOP], the Alzheimer's Association ‐ Part the Cloud: Translational Research Funding for Alzheimer's Disease [18PTC‐19‐602325] and the Alzheimer's Association ‐ GAAIN Exploration to Evaluate Novel Alzheimer's Queries [GEENA‐Q‐19‐596,282]).

## Author Roles

(1) Research project: A. Conception, B. Organization, C. Execution; (2) Statistical Analysis: A. Design, B. Execution, C. Review and Critique; (3) Manuscript: A. Writing of the First Draft, B. Review and Critique.

S.D.P.:1A, 1B, 1C; 2A, 2B; 3A

R.F.: 1B, 1C; 3B

P.C.: 1C

A.F.: 1C; 3B

R.A.E.: 3B

C.S.: 2A, 2C; 3B

M.R.: 3B

S.L.S.: 1A, 1B; 2C; 3A

M.O.: 1A, 1B, 3A

## Supporting information


**Appendix S1** Supporting information

## Data Availability

Data are available upon request.
