# Supplementary material for: Altered Medial Prefrontal Connectivity in Parkinson's Disease Patients with Somatic Symptoms
Source: Mov Disord. 2022 Aug 22;37(11):2226–35. doi: 10.1002/mds.29187 (PMC9804911; doi:10.1002/mds.29187)
Supplement: Supplementary file 1 — Appendix S1 Supporting information [file MDS-37-2226-s001.docx]

**Structural MRI analysis**

The preprocessing steps encompassed motion correction, removal of non-brain tissue using a hybrid watershed/surface deformation procedure, automated Talairach transformation, segmentation of the subcortical WM and deep GM volumetric structures, and intensity normalization, tessellation of the gray matter white matter boundary, automated topology correction, and surface deformation. This last step was computed following intensity gradients to optimally place the gray/white and gray/cerebrospinal fluid borders at locations where the greatest shift in intensity defines the transition to the other tissue class. Once the cortical models were completed, a number of deformable procedures were performed for further data processing and analysis, including surface inflation, registration to a spherical atlas based on individual cortical folding patterns to match cortical geometry across subjects, parcellation of the cerebral cortex into units with respect to gyral and sulcal structure, and creation of a variety of surface-based data including maps of curvature and sulcal depth. This method uses intensity and continuity information from the entire three-dimensional MR volume in segmentation and deformation procedures to produce representations of cortical thickness, calculated as the closest distance from the gray/white boundary to the gray/CSF boundary at each vertex on the tessellated surface.

**
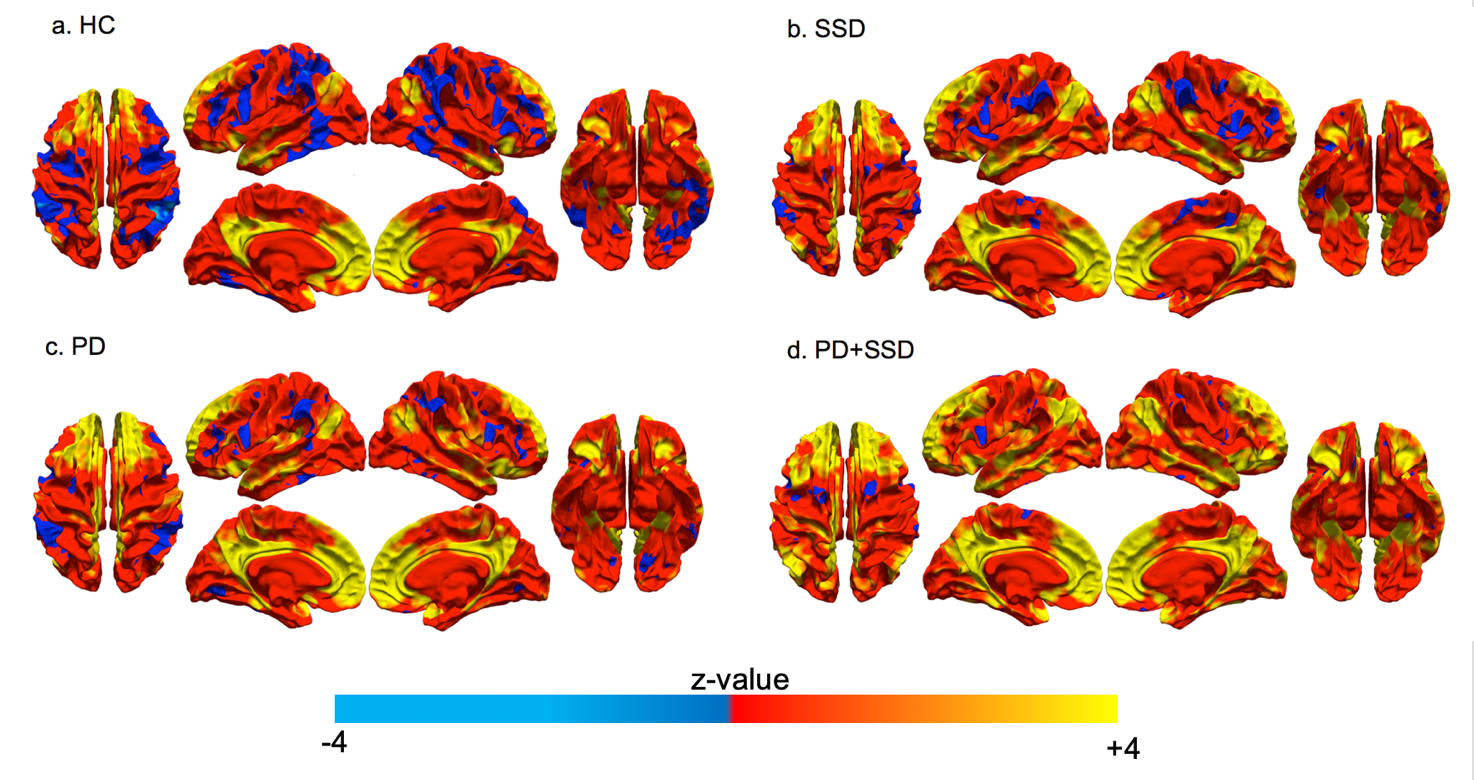
**

**Supplementary figure 1.** Maps of functional connectivity of 1H-MRS-VOI with the rest of the brain in HC (panel A), SSD (panel B), PD (panel C), and PD+SSD (panel D). Clusters changing from red to yellow and dark blue to blue indicate positive or negative connectivity, respectively.

**
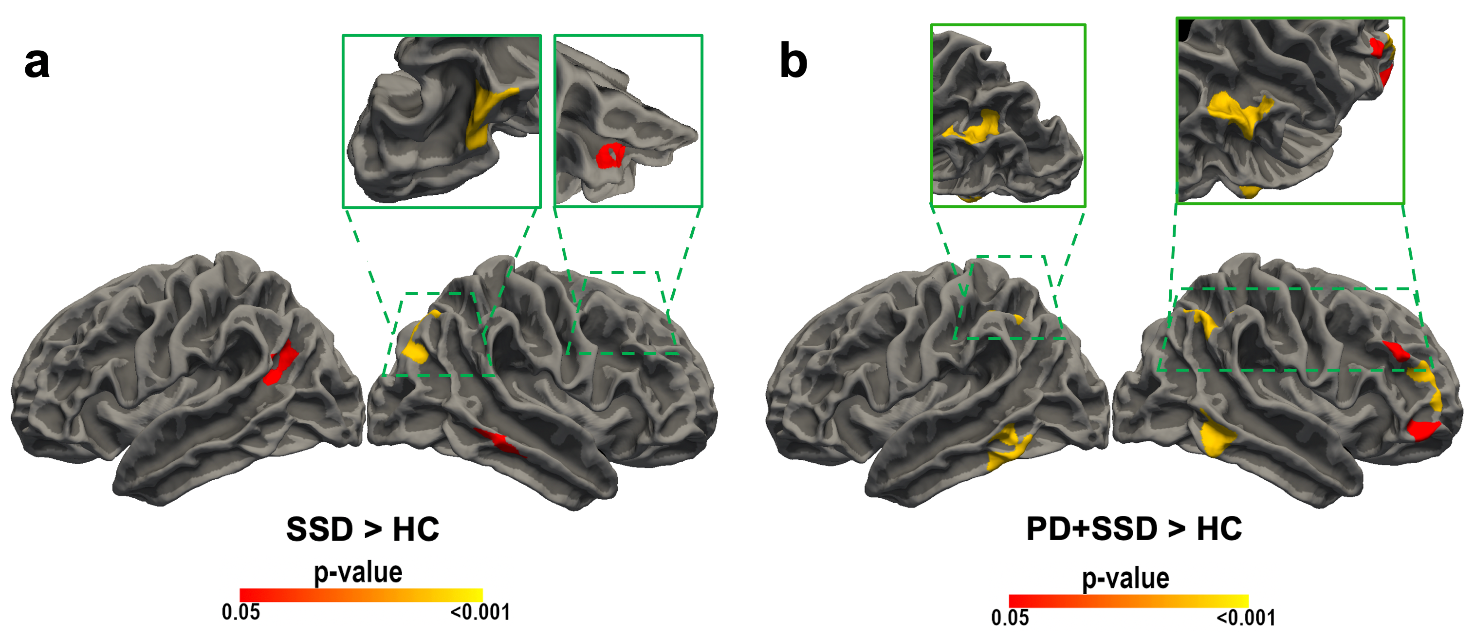
**

**Supplementary figure 2.** Statistical maps showing the difference among SSD or PD+SSD patients with HC subjects on functional connectivity of the 1H-MRS-VOI centered on the medial prefrontal cortex. Panel A shows the comparison of SSD patients with HC subjects. Panel B shows the comparison of PD+SSD patients with HC subjects. Clusters changing from red to yellow indicate increased connectivity. The figure depicts areas with a cluster-wise probability below the corrected p-value of 0.05. *Keys:* HC, healthy controls; PD+SSD, patients with Parkinson's disease without somatic symptom disorder; PD, patients without Parkinson's disease without somatic symptom disorder; SSD, patients with somatic symptom disorder unaffected by any neurological or other psychiatric condition.

**Supplementary Table 1.** Symptoms Questionnaire-subscale items for each group

|  | HC | SSD | PD | SSD+PD |
| --- | --- | --- | --- | --- |
| Anger, Hostility | 2.5±1.0 | 4.7±2.8 | 3.5±2.3 | 4.7±2.9 |
| Anxiety | 3.4±1.3 | 5.2±2.5 | 4.3±2.4 | 5.3±3.6 |
| Contentment | 0.5±0.6 | 1.6±1.6 | 0.6±0.8 | 1.6±2.1 |
| Depression | 2.6±0.9 | 4.4±3.3 | 2.3±1.0 | 4.8±4.1 |
| Friendliness | 0.4±0.6 | 0.8±1.1 | 0.4±0.6 | 0.8±0.9 |
| Physical Wellbeing | 0.5±0.9 | 3.2±2.1* | 1.7±1.3 | 3.7±1.9* |
| Relaxation | 0.6±0.6 | 1.1±1.0 | 0.6±0.6 | 1.2±1.2 |
| Somatic | 2.6±0.9 | 10.5±4.7* | 4.4±2.5 | 11.1±4.1* |

Values are expressed as means ± standard deviations (SD). Scores significantly different among groups were marked with an asterisk.

*Key:* HC, healthy control subjects; SSD, individuals with Somatic Symptom Disorder who did not show other neurological or psychiatric conditions; PD, Parkinson's Disease patients without Somatic Symptom disorder; SSD+PD, Parkinson's Disease patients with Somatic Symptom Disorder.

**Supplementary table 2.** Location of target regions expressing functional connectivity changes of the mPFC with cortex

| Statistics | Max | VtxMax | Size (mm^2^) | Talairach coordinate of the maximum | | | CWP | NVtxs | Cortical target localization | |
| --- | --- | --- | --- | --- | --- | --- | --- | --- | --- | --- |
|  |  |  |  |  |  |  |  |  | Desikan's Atlas region | Network |
|  |  |  |  | X | Y | Z |  |  |  |  |
| SSD vs. noSSD | 6.4545 | 78565 | 1239.7 | 35 | -74.2 | 40 | 0.0002 | 2425 | R-inferiorparietal | DMN/EAN |
|  | 6.9001 | 24560 | 775.53 | 47.9 | 29.5 | 27.6 | 0.0002 | 1288 | R-rostralmiddlefrontal | EAN |
|  | 4.5203 | 86011 | 613.17 | 8.7 | -46.3 | 12.7 | 0.0002 | 1572 | R-isthmuscingulate | DMN |
|  | 4.1732 | 45019 | 468.47 | 7.2 | -69.9 | 42.9 | 0.0002 | 1104 | R-precuneus | DMN |
|  | 6.4925 | 92604 | 397.52 | 35.1 | 50.7 | 6.8 | 0.0002 | 589 | R-rostralmiddlefrontal | EAN |
|  | 5.6108 | 44320 | 377.41 | 29.3 | 16.3 | 44.4 | 0.0004 | 773 | R-caudalmiddlefrontal | EAN |
|  | 5.4541 | 162586 | 332.17 | 62.2 | -42.7 | -7.7 | 0.0008 | 517 | R-middletemporal | DMN/EAN |
|  | 5.7338 | 50469 | 319.17 | 35 | -31.3 | 39.6 | 0.001 | 918 | R-supramarginal | DMN |
|  | 4.7043 | 147759 | 290.67 | 52.3 | -51.7 | 41.4 | 0.003 | 652 | R-inferiorparietal | DMN/EAN |
|  | 4.3672 | 123144 | 215.73 | 17.5 | 42.5 | 38 | 0.02346 | 336 | R-superiorfrontal | DMN |
|  | 4.7093 | 100592 | 189.09 | 44.1 | 44.3 | -10.9 | 0.04038 | 270 | R-parsorbitalis | EAN/limbic |
|  | 5.9976 | 18394 | 1245.98 | -36.1 | -56.3 | 36.1 | 0.0002 | 2644 | L-inferiorparietal | DMN/EAN |
|  | 4.3436 | 106334 | 398.26 | -40.8 | 27.4 | 25.7 | 0.0002 | 699 | L-rostralmiddlefrontal | EAN |
|  | 5.4942 | 137079 | 324.47 | -57.5 | -46.7 | -9.3 | 0.002 | 501 | L-middletemporal | DMN/EAN |
|  | 3.5039 | 62077 | 211.84 | -6.7 | -40.9 | 32.2 | 0.02227 | 480 | L-isthmuscingulate | DMN |
|  | 4.0815 | 85614 | 204.51 | -57.1 | -50.6 | 30 | 0.02544 | 472 | L-supramarginal | DMN |
|  | 4.3109 | 76160 | 202.3 | -30.7 | 7 | 54.7 | 0.02682 | 353 | L-caudalmiddlefrontal | EAN |
|  | 3.9347 | 142859 | 191.72 | -13.1 | -62.6 | 25.3 | 0.03685 | 356 | L-precuneus | DMN |
| SSD x PD | -6 | 163712 | 183.89 | 55.1 | -18.6 | -23.4 | 0.04488 | 281 | R-middletemporal | DMN |
| PD+SSD vs. PD | 5.292 | 92603 | 222.55 | 34.4 | 50.2 | 6.3 | 0.02069 | 323 | R-rostralmiddlefrontal | EAN |
|  | 4.0824 | 78235 | 187.3 | -36.6 | -60.4 | 39 | 0.04136 | 427 | L-inferiorparietal | EAN |
| SSD vs. HC | 6.3388 | 78567 | 521.34 | 35.1 | -74.6 | 39.9 | 0.0002 | 893 | R-inferiorparietal | DMN |
|  | 4.7677 | 34522 | 270.02 | 31.3 | 16.5 | 46.2 | 0.00559 | 545 | R-caudalmiddlefrontal | DMN |
|  | 5.3157 | 54352 | 206.72 | 62.5 | -31.3 | -15.1 | 0.02721 | 347 | R-middletemporal | DMN |
|  | 4.1086 | 78129 | 302.68 | -49.7 | -53.9 | 22.4 | 0.0034 | 678 | L-supramarginal | DMN |
| PD+SSD vs. SSD | 6.9176 | 78468 | 1097.97 | 43.6 | -58.1 | 44.6 | 0.0002 | 2501 | R-inferiorparietal | EAN |
|  | 5.8922 | 93703 | 489.86 | 35.1 | 48.2 | 16.3 | 0.0002 | 715 | R-rostralmiddlefrontal | EAN |
|  | 6.481 | 53480 | 452.22 | 56.9 | -47.2 | -13.8 | 0.0002 | 706 | R-inferiortemporal | EAN |
|  | 5.2593 | 45645 | 211.91 | 44.2 | 43.5 | -9.9 | 0.02504 | 292 | R-parsorbitalis | EAN |
|  | 5.1896 | 152222 | 198.64 | 43.8 | 33 | 28.1 | 0.03391 | 325 | R-rostralmiddlefrontal | EAN |
|  | 6.6194 | 78221 | 817.17 | -35.7 | -56.3 | 36 | 0.0002 | 2048 | L-inferiorparietal | EAN |
|  | 5.0483 | 161926 | 561.85 | -57.3 | -47.8 | -8.9 | 0.0002 | 810 | L-middletemporal | EAN |

*Keys:* CWP, cluster-wise p-value (this is the p-value of the cluster); HC, healthy controls; DMN, default mode network; EAN=executive attention network; PD+SSD, patients with Parkinson's disease without somatic symptom disorder; Max, the maximum -log_10_ (p-value) in the cluster; NVtxs, number of vertices in the cluster; PD, patients without Parkinson's disease without somatic symptom disorder; Size, surface area (mm^2^) of cluster; SSD, patients with somatic symptom disorder unaffected by any neurological or other psychiatric condition; VtxMax, the vertex number at the maximum.
